# Combined effect of high hydrostatic pressure and ultraviolet radiation on quality parameters of refrigerated vacuum-packed tilapia (*Oreochromis niloticus*) fillets

**DOI:** 10.1038/s41598-018-27861-9

**Published:** 2018-06-22

**Authors:** Maria Lúcia Guerra Monteiro, Eliane Teixeira Mársico, Sérgio Borges Mano, Thiago da Silveira Alvares, Amauri Rosenthal, Mosar Lemos, Elisa Ferrari, Cesar Aquiles Lázaro, Carlos Adam Conte-Junior

**Affiliations:** 1 Department of Food Technology, Universidade Federal Fluminense, 24230-340 Rio de Janeiro, Brazil; 2 Chemistry Institute, Universidade Federal do Rio de Janeiro, 21941-909 Rio de Janeiro, Brazil; 3 Nutrition Institute, Universidade Federal do Rio de Janeiro, 27979-000 Rio de Janeiro, Brazil; 4 Embrapa Food Technology, 23020-470 Rio de Janeiro, Brazil; 5 Facultad de Medicina Veterinaria, Universidad Nacional Mayor de San Marcos, 03-5137 Lima, Peru; 6 National Institute of Health Quality Control, Fundação Oswaldo Cruz, 21040-900 Rio de Janeiro, Brazil

## Abstract

This study investigated the effects of high hydrostatic pressure (HHP) and ultraviolet radiation (UV-C), individually and combined, on the physical, chemical and bacterial parameters of Nile tilapia (*Oreochromis niloticus*) fillets stored at 4 °C for 14 days. Tilapia fillets were divided into four groups: control (untreated samples), UV-C, HHP, and UV-C combined with HHP (UV-C+HHP); UV-C was applied at dose of 0.103 ± 0.002 J/cm^2^, and HHP at a pressure of 220 MPa for 10 min at 25 °C. All samples were analyzed for total aerobic mesophilic count (TAMC), total aerobic psychrotrophic count (TAPC), Enterobacteriaceae count, pH, lipid oxidation, total volatile basic nitrogen (TVB-N), ammonia (NH_3_), and biogenic amines. Although UV-C accelerated (*P* ≤ 0.05) the formation of cadaverine, both UV-C and HHP, alone or together, retarded bacterial growth and delayed the increase (*P* ≤ 0.05) in pH, TVB-N, NH_3_ and biogenic amines during refrigerated storage, extending the shelf life of refrigerated tilapia fillets at least 2.5 times considering the TAMC counts. Lipid oxidation was unaffected (*P* > 0.05) by UV-C radiation, and decreased (*P* ≤ 0.05) by HHP and UV-C+HHP. HHP alone or combined with UV-C showed higher potential benefits for tilapia fillets preservation considering the positive influence on cadaverine levels and lipid oxidation.

## Introduction

Fish are rich in essential amino acids and polyunsaturated fatty acids^1^; however, shelf life is a limiting factor due to their high perishability^2,3^. The rapid deterioration of fish is attributed to intense action of autolytic enzymes and rapid microbial growth due to the nutrient composition, resulting in several metabolites and quality loss^4,5^. The United Nations Food and Agriculture Organization has encouraged studies of conservation methods to control microbial growth and ensure food safety^6^.

High hydrostatic pressure (HHP) is a non-thermal technology used to extend the shelf life of perishable foods while preserving their original properties, depending on the type of food and pressure applied^7^. HHP either inactivates or produces sublethal injury to most microbial vegetative cells; and pressures between 200–300 MPa may affect the growth and reproduction of most pathogenic and spoilage microorganisms^8^. HHP has been recommended to reduce pathogens in seafood^9^, and several products treated with HHP are commercially available, such as dry-cured ham in Germany, Italy and Spain; chicken (sausages, strips and breast slices) in the USA; and ready-to-eat meat-based meals with rice, pasta or vegetables in Canada^10^. Although HHP is already used industrially, fish and fish products are not included in the FDA list. The ideal parameters (pressure level, time, temperature) need to be determined for each food, in order to extend the shelf life while retaining the nutritional and sensory qualities. The effectiveness of HHP varies with the type of matrix and with the microorganisms (genus; specie and strain; positive or negative Gram; spore or vegetative cells; and growth phases – lag, log or stationary phase)^7,11^.

Another emerging non-thermal technology is ultraviolet radiation type C (UV-C, wavelengths of 200−280 nm), which acts directly on the microbial DNA and reduces the bacterial load in foods^12^. UV-C radiation is approved by the FDA for surface decontamination of food products^13^ and has several advantages such as ease of implementation, low cost, lack of toxic wastes, and maintenance of the nutritional and sensory characteristics of the products when appropriate doses are applied^12^. The effect of UV-C depends mainly on the type of food and dose utilized^12,14,15^. Therefore, studies of UV-C utilization in previously uninvestigated foods such as tilapia fillets may help to encourage its industrial application.

Although previous studies have demonstrated the effects of the HHP and UV-C methods on improving the quality of several fish species^5,16–18^, none has evaluated the combined effect of HHP and UV-C on the shelf life of refrigerated fish. In addition, knowledge of UV-C technology applied to fish species is still sparse. The present study investigated the effect of HHP and UV-C, individually and combined, on physical, chemical and bacterial parameters of tilapia (*Oreochromis niloticus*) fillets stored at 4 °C for 14 days.

## Materials and Methods

### Experimental design

One hundred and forty tilapia fillets (120.0 g ± 5.1 g, mean ± SD) packed in low-density polyethylene bags were obtained from a fish farm in Rio de Janeiro, Brazil (22°33′58.3″S 042°41′48.2″W) and transported in ice to the laboratory within 2 h. Then, the fillets were individually vacuum-packed (Engevac^®^, São Paulo, Brazil) and randomly separated into four groups: control (untreated samples), ultraviolet radiation (UV-C; 0.103 ± 0.002 J/cm^2^, mean ± SD), high hydrostatic pressure (HHP; 220 MPa for 10 min at 25 °C), and UV-C radiation combined with HHP (UV-C+HHP) in the same conditions previously described. The control group was composed of 20 packages (4 storage times × 5 replicates), while the UV-C, HHP and UV-C+HHP groups each comprised 40 packages (8 storage times × 5 replicates). The tilapia fillets were stored at 4 °C, and UV-C and HHP processing were performed. All treatments were analyzed for total aerobic mesophilic count (TAMC), total aerobic psychrotrophic count (TAPC), Enterobacteriaceae count, pH, lipid oxidation, total volatile basic nitrogen (TVB-N), ammonia (NH_3_), and biogenic amines. The tests continued until the samples reached 7.0 log cfu (colony-forming units)/g for TAMC, the point at which fish is considered unacceptable for consumption according to International Commission on Microbiological Specifications for Foods^19^. This limit was considered the bacteriological criterion to express the shelf life of tilapia fillets during the storage period. Bacteriological, physical and chemical parameters of the control samples were determined on days 0, 1, 2, and 4; and in the HHP, UV-C, and UV-C+HHP samples on days 0, 1, 2, 4, 6, 9, 11, and 14. On each day of storage, one fillet of each treatment was analyzed for parameters aforementioned. The entire experiment was repeated five times (n = 5). All analyses were performed in duplicate.

### UV-C radiation exposure

The UV-C equipment consisted of six UV-C lamps of 30 W and six UV-C lamps of 55 W arranged in an interleaved manner, as designed by Lázaro *et al*.^20^. Before UV-C application, all lamps were turned on, and after 15 min the UV-C intensity stabilized. The distance between the fillet surface and the UV-C lamps was 14 cm. Individual samples were placed in the center of the UV-C apparatus, and the intensities were controlled by a UV radiometer (MRUR-203, Instrutherm Ltda., São Paulo, Brazil) which was covered with vacuum packaging and placed beside the samples to be irradiated. The dose was monitored every 5 s until reaching 0.103 ± 0.002 J/cm^2^ when the lamps were turned off. The dose of 0.103 J/cm^2^ was chosen due to its effectiveness in delaying the doubling time of microorganisms while maintaining the physical and chemical parameters almost intact, in species of fish^21^.

### High hydrostatic pressure processing

High-pressure processing was carried out in a laboratory-scale pressurizer (Stansted Fluid Power, model S-FL-850-9-W, Essex, UK). The high hydrostatic pressure apparatus contained a perforated stainless-steel sample holder cylinder where the samples were introduced, and the chamber was hermetically sealed prior to pressurization. The pressure-transmitting liquid utilized was 70% ethanol. The HHP equipment was programmed to 220 MPa for 10 min at 25 °C, based on previous studies^8,16,22^, in order to obtain the antimicrobial effect with only minor changes in the physical and chemical parameters of tilapia fillets.

### Bacteriological analyses

Aliquots of 25 g were aseptically weighed in a sterile stomacher bag (Labplas Inc., Sainte-Julie, Québec, Canada) and 225 mL of sterile peptone saline was added. The bag content was homogenized for 2 min in a stomacher (Stomacher 80, Seward, London, UK), and serial dilutions were inoculated by the pour-plate technique, into Petri dishes containing a specific culture medium. TAMC and TAPC was enumerated on plate-count agar (PCA, Merck) after incubation at 37 °C for 48 h^23^ and at 10 °C for 7 days^24^, respectively. Enterobacteriaceae were cultured on Violet-Red-Bile-Glucose agar (VRBG-agar, Merck) and counted following incubation at 35 °C for 24 h^25^. The results were expressed as log cfu per gram.

### Fish pH

The pH was measured in 1 g of homogenized sample plus 9 mL of distilled water, using a digital pH meter (K39-1014B, Kasvi, Paraná, Brazil)^26^. Before the pH measurements, buffered solutions at pH 4 and 7 were used to calibrate the pH meter.

### Lipid oxidation

Lipid oxidation was determined through thiobarbituric acid-reactive substances (TBARS), following the method of Yin *et al*.^27^. The absorbance values were measured at 532 nm, using a UV-1800 spectrophotometer (Shimadzu, Kyoto, Japan). The results were expressed as mg of malonaldehyde (MDA)/kg of fish meat from a standard curve (R^2^ = 0.997) made with seven different MDA concentrations raging from 1 to 500 µmol.

### Total volatile basic nitrogen (TVB-N) and ammonia

TVB-N was determined through Conway’s microdiffusion method, according to protocol established by the Association of Official Analytical Chemists^28^. Results were expressed as mg TVB-N/100 g. Ammonia was quantified by the colorimetric method of McCullough^29^ modified by Rodrigues *et al*.^5^, utilizing a UV-1800 spectrophotometer (Shimadzu, Kyoto, Japan). Results were expressed in µg NH_3_/g.

### Biogenic amines

The biogenic amines (BAs) were evaluated with a high-performance liquid chromatography system (Shimadzu, Kyoto, Japan), with a CBM-20A controller composed of a LC-20AT pump, SPD-M20A diode-array detector, CTO-20A oven and SIL-20AC autosampler^30^. A Spherisorb ODS-2 C18 column (15 × 0.46 cm id, 5 μm particle size) was utilized as the stationary phase, and an acetonitrile:water mixture (42:58, v/v) was used as the mobile phase under isocratic conditions. The injection volume was 20 µL, the flow rate of the mobile phase was 1 mL/min, the temperature of column was set to 40 °C, and the signals were detected at 254 nm.

### Statistical analyses

A total of 140 tilapia fillets (4 fillets for control group and 8 fillets for each UV-C, HHP, and UV-C + HHP group × 5 replicates) were used (n = 5). To identify differences in the pH, lipid oxidation, TVB-N, ammonia and BAs between control, UV-C radiation (0.103 ± 0.002 J/cm^2^), HHP processing (220 MPa for 10 min at 25 °C), and UV-C+HHP over the days of storage (0, 1, 2, 4, 6, 9, 11, and 14), two-way ANOVA was used. When a significant *F* was found, additional post-hoc tests with Tukey adjustment were performed. Statistical significance was set at the 0.05 level of confidence, and all analyses were performed using XLSTAT software, version 2012.6.08 (Addinsoft, New York, NY, USA). The bacterial growth curves were obtained through predictive primary model designed by Baranyi & Roberts^31^, utilizing the DMFit program, version 2.0 (Institute of Food Research, Norwich, UK) in order to evaluate the effect of each treatment applied in the bacterial growth parameters (lag, log and stationary phases) through one-way ANOVA with Tukey post-hoc test (*P* ≤ 0.05).

## Results and Discussion

### Bacteriological evaluation

**Figure 1:**
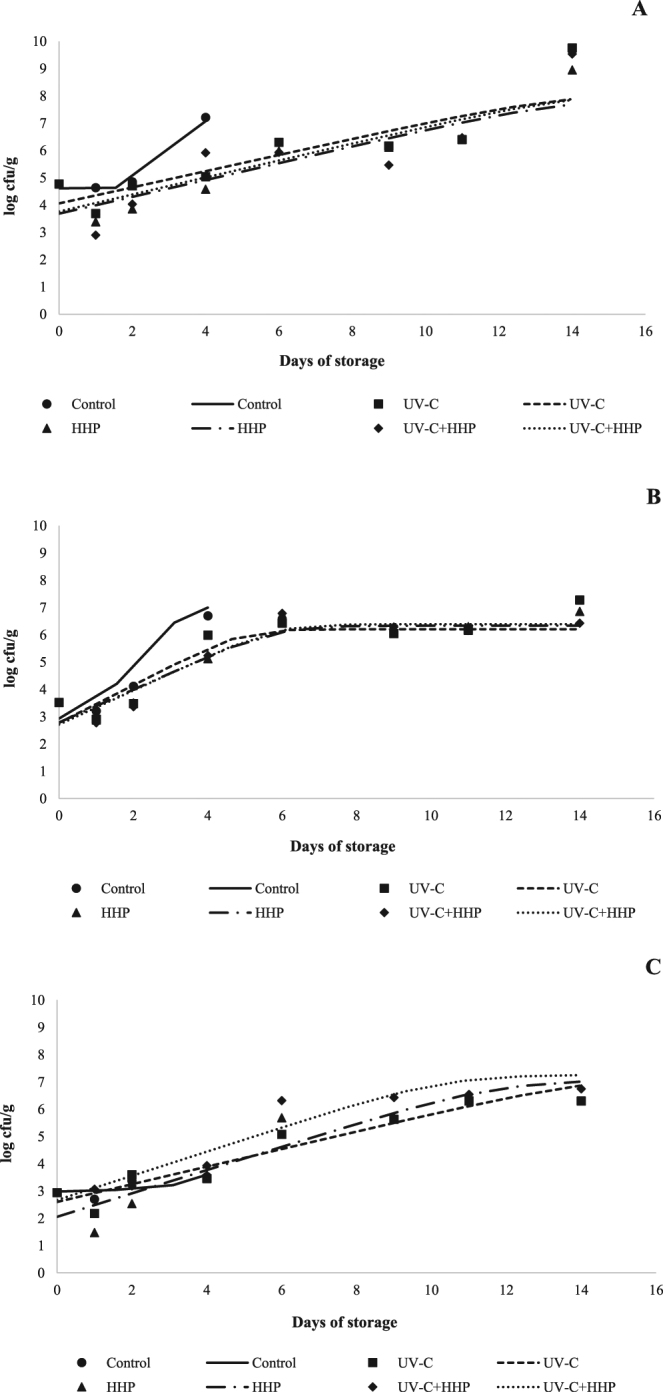
Total aerobic mesophilic count (**A**), Total aerobic psychrotrophic count (**B**), and Enterobacteria count (**C**) in Nile tilapia (*Oreochromis niloticus*) fillets exposed to UV-C, HHP and UV-C+HHP processes under refrigeration storage (4 °C) for 14 days. Control – untreated fillets; UV-C – fillets treated with ultraviolet radiation type C (0.103 ± 0.002 J/cm^2^); HHP – fillets submitted to high hydrostatic pressure (220 MPa for 10 min at 25 °C); UV-C+HHP – fillets subjected to high hydrostatic pressure (220 MPa for 10 min at 25 °C) and ultraviolet radiation type C (0.103 ± 0.002 J/cm^2^). Symbols indicates the real average values (n = 5) and lines represents the fitted values by predictive primary model designed by Baranyi & Roberts^31^.

The TAMC, TAPC and Enterobacteriaceae counts are shown in Fig. 1A–C. For all treatments, the initial logs (day 0) of the TAMC, TAPC and Enterobacteriaceae counts were 4.77 ± 0.01, 3.52 ± 0.02 and 2.94 ± 0.02 log cfu/g, respectively. Control samples exceed the limit of 7.0 log cfu/g for TAMC^19^ on storage day 4, while tilapia fillets treated with HHP, UV-C and UV-C+HHP reached this limit on storage day 14. The TAPC count exceeded the recommended limit on days 4 and 14 in the control and UV-C samples, respectively, while HHP and UV-C+HHP fillets did not exceed this limit for TAPC during storage. The presence of Enterobacteriaceae in fish is related mainly to cross contamination during fish processing such as filleting^32^, however, in our study, the initial Enterobacteriaceae counts were below 3 cfu/g indicating that the tilapia fillets used were in good quality^19^. A lag phase was observed in the control samples, except for TAPC. In addition, for all bacterial groups, the samples submitted to the HHP and UV-C processes, combined or alone, showed longer doubling times (*P* ≤ 0.05) and fewer colonies in the stationary phase (*P* ≤ 0.05) than the untreated samples (Table 1), explaining the greater bacteriological quality for HHP, UV-C and UV-C+HHP samples. The lag phase in the control samples can be attributed to changes in temperature^33^. The Nile tilapia is a warm-water species with optimum growth from 25 to 31 °C^34^, and mesophilic bacteria require an adaption period for growth under refrigeration up to 15 °C; whereas psychrotrophic bacteria such as *Pseudomonas* spp. grow rapidly below 15 °C^35^. The Enterobacteriaceae group is composed by Gram negative bacteria, which are more sensitive to low temperature than Gram positive bacteria^36,37^.

**Table 1:** Bacterial growth parameters of Nile tilapia (*Oreochromis niloticus*) fillets submitted to UV-C, HHP and UV-C+HHP stored at 4 °C for 14 days.

Treatments^*^					
Microorganism^¥^	Parameter^#^	Control	UV-C	HHP	UV-C+HHP
TAMC	Lag	2.17 ± 0.00^a^	0.00 ± 0.00^b^	0.00 ± 0.00^b^	0.00 ± 0.00^b^
	Log	0.76 ± 0.01^b^	1.00 ± 0.02^a^	0.96 ± 0.03^a^	0.95 ± 0.03^a^
	NC	8.46 ± 0.02^a^	8.00 ± 0.01^d^	8.14 ± 0.02^c^	8.23 ± 0.02^b^
	R^2^	0.862	0.851	0.847	0.858
TAPC	Lag	0.00 ± 0.00^a^	0.00 ± 0.00^a^	0.00 ± 0.00^a^	0.00 ± 0.00^a^
	Log	0.36 ± 0.01^b^	1.02 ± 0.03^a^	0.96 ± 0.05^a^	0.99 ± 0.01^a^
	NC	7.22 ± 0.01^a^	6.93 ± 0.11^b^	6.95 ± 0.07^b^	6.75 ± 0.35^b^
	R^2^	0.802	0.890	0.813	0.890
Enterobacteria	Lag	3.86 ± 0.00^a^	0.00 ± 0.00^b^	0.00 ± 0.00^b^	0.00 ± 0.00^b^
	Log	0.62 ± 0.00^c^	0.93 ± 0.00^a^	0.69 ± 0.03^b^	0.67 ± 0.01^b^
	NC	7.78 ± 0.00^a^	7.07 ± 0.02^c^	7.30 ± 0.03^b^	7.25 ± 0.02^b^
	R^2^	0.992	0.903	0.901	0.911

^¥^TAMC - Total aerobic mesophilic count; TAPC - Total aerobic psychrotrophic count.

^#^Lag – lag phase (days); Log – log phase (hours); NC – number of colonies in the stationary phase (log cfu/g).

^*^Control – untreated fillets; UV-C – fillets treated with ultraviolet radiation type C (0.103 ± 0.002 J/cm^2^); HHP – fillets submitted to high hydrostatic pressure (220 MPa for 10 min at 25 °C); UV-C+HHP – fillets subjected to high hydrostatic pressure (220 MPa for 10 min at 25 °C) and ultraviolet radiation type C (0.103 ± 0.002 J/cm^2^).

Results are expressed as means ± standard deviation.

^a–d^Different letters in the same row indicate significant differences (*P* ≤ 0.05).

HHP, an emerging non-thermal technology, acts directly on the membranes, morphology and biochemical reactions of bacterial cells, inactivating pathogens and spoilage microorganisms^38^. UV-C radiation, also an emerging non-thermal technology, promotes surface decontamination of the foods by inducing thymine-cytosine cross-linking, resulting in impairment of microbial DNA^12^. Both the HHP and UV-C technologies can reduce the initial bacterial load, extending the shelf life; however, they can also increase the nutrient bioavailability and affect the competitive environment, allowing rapid growth of microorganisms that recover from a sublethal effect and do not require an adaptation period^12,20,38,39^. On the other hand, bacterial cells damaged by HHP and UV-C grow more slowly than intact cells^16,40^, although the effects of HHP and UV-C on the behavior of microorganisms can vary according to the type of food and the pressure level or UV-C dose applied^7,12,14^. Bottino *et al*.^40^ and Molina *et al*.^17^ observed an increased shelf life in hybrid tambacu (*Colossoma macropomum* × *Piaractus mesopotamicus*) fillets and cultured sea bass (*Dicentrarchus labrax*) fillets treated with UV-C radiation and stored at 4 °C, respectively. In contrast, in spite of the lag phase in mesophilic bacteria caused by UV-C exposure, no effect on the shelf life of rainbow-trout (*Oncorhynchus mykiss*) fillets at 4 °C was reported by Rodrigues *et al*.^5^. In agreement with our results, HHP in similar conditions prolonged the shelf life of refrigerated red mullet (*Mullus surmelutus*) fillets^22^ and rainbow-trout fillets stored at 4 °C^41^.

### Fish pH

**Figure 2:**
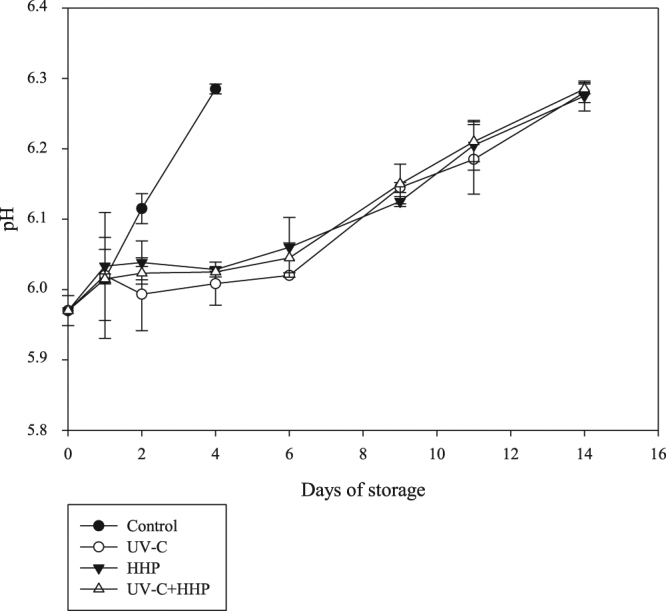
pH in Nile tilapia (*Oreochromis niloticus*) fillets exposed to UV-C, HHP and UV-C+HHP processes under refrigeration storage (4 °C) for 14 days. Control – untreated fillets; UV-C – fillets treated with ultraviolet radiation type C (0.103 ± 0.002 J/cm^2^); HHP – fillets submitted to high hydrostatic pressure (220 MPa for 10 min at 25 °C); UV-C+HHP – fillets subjected to high hydrostatic pressure (220 MPa for 10 min at 25 °C) and ultraviolet radiation type C (0.103 ± 0.002 J/cm^2^). Error bars indicate the standard deviation of the mean (n = 5).

The pH of tilapia fillets on day 0 was 5.98 ± 0.02 (Fig. 2). The pH of live fish is close to neutrality; however, it decreases quickly after fish death due to glycogen conversion to lactic acid, ranging from 6.0 to 6.8 in most fish species^42^. Similar initial pH levels were previously reported in fillets of tilapia and other freshwater fish species such as rainbow trout and hybrid tambacu^5,40,42^. Fish pH increased (*P* ≤ 0.05) after day 1 for control samples, and after day 6 for UV-C, HHP, and UV-C+HHP samples until the end of the refrigerated storage period. The increase of pH levels during storage is associated with the accumulation of alkaline compounds from microbial activity in the post-rigor period^5^. A similar pH trend was noted in tilapia fillets under refrigerated storage^4,42,43^. The pH levels increased quickly (*P* *≤* 0.05) in the control samples compared to the HHP, UV-C and UV-C+HHP samples. After the first day of storage, the HHP, UV-C and UV-C+HHP samples showed lower (*P* ≤ 0.05) pH levels than their control counterparts, and no difference (*P* > 0.05) was observed among samples treated with HHP and UV-C, individually or combined, during the entire refrigerated-storage period. Both HHP and UV-C reduce bacterial growth, and therefore control the formation of alkaline compounds during the degradation process^5,41^. In agreement with our findings, Erkan & Uretener^44^ and Günlü *et al*.^41^ observed a slower increase of pH levels in sea bream (*Sparus aurata*) and rainbow-trout fillets, respectively, under similar HHP and storage-temperature conditions. Molina *et al*.^17^ reported the same effect on pH levels in refrigerated sea-bass fillets exposed to UV-C.

### Lipid oxidation

**Figure 3:**
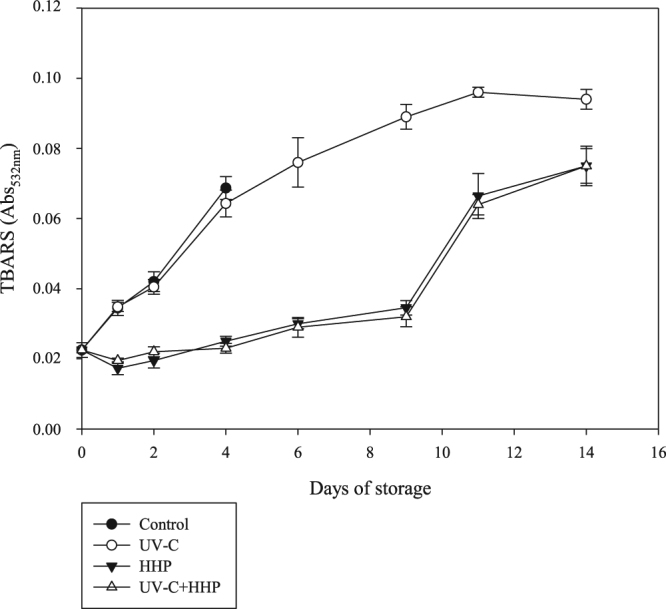
Lipid oxidation in Nile tilapia (*Oreochromis niloticus*) fillets exposed to UV-C, HHP and UV-C+HHP processes under refrigeration storage (4 °C) for 14 days. Control – untreated fillets; UV-C – fillets treated with ultraviolet radiation type C (0.103 ± 0.002 J/cm^2^); HHP – fillets submitted to high hydrostatic pressure (220 MPa for 10 min at 25 °C); UV-C+HHP – fillets subjected to high hydrostatic pressure (220 MPa for 10 min at 25 °C) and ultraviolet radiation type C (0.103 ± 0.002 J/cm^2^). Error bars indicate the standard deviation of the mean (n = 5).

Variations in TBARS levels are shown in Fig. 3. Lipid oxidation increased (*P* ≤ 0.05) during the entire storage period in all treatments. The increase in TBARS levels during storage is due to the action of endogenous enzymes such as lipoxygenase^45^ and pro-oxidant agents such as free iron released from protein degradation^46^. A similar increasing trend was observed in Nile tilapia stored under refrigeration^4,42,43^. Nevertheless, the increase of the lipid oxidation was less pronounced (*P* ≤ 0.05) in HHP and UV-C+HHP than control and UV-C samples. The acceptability limit for TBARS is equal to 2 mg of MDA/kg of meat^47^. While control and UV-C samples exceed this limit on day 4, HHP and UV-C+HHP samples had values above 2 mg of MDA/kg of meat only on day 11 of refrigerated storage.

The HHP and UV-C+HHP samples showed lower (*P* ≤ 0.05) TBARS levels compared to the control and UV-C samples during the entire storage period with no difference (*P* > 0.05) between the control and UV-C samples up to 4 storage days, and between HHP and UV-C+HHP samples throughout the storage time. The effect of the HHP on fish muscle depends mainly on the combination of the muscle composition (lipid and non-lipid fractions) and the HHP processing conditions^48,49^. Although the effect of HHP on lipid oxidation is not fully understood^48^, inactivation of lipoxygenase by HHP treatment was previously observed in a crude extract from silver carp (*Hypophthalmichthys molitrix*), depending on the time and temperature interaction^18^. In agreement with our data, Erkan *et al*.^22^ reported that HHP, under the same conditions as the present study (220 MPa for 10 min at 25 °C), decreased TBARS levels in refrigerated rainbow trout. Erkan & Uretener^44^ observed lower TBARS levels in sea bream (*Sparus aurata*) after HHP at 250 MPa for 10 min at 7 °C and at 330 MPa for 10 min at 25 °C.

The lack of influence of UV-C radiation on lipid oxidation of tilapia fillets stored under refrigeration can be attributed to the low total lipid content of tilapia, approximately 1.7%^50^. Moreover, the low UV-C dose and absence of oxygen from vacuum packaging applied in the present study may have contributed to no effect on lipid oxidation. Similarly to our findings, no change in lipid oxidation by UV-C exposure at a similar dose was detected in rainbow-trout fillets^5^ or in hybrid tambacu fillets^40^. In addition, Molina *et al*.^17^ observed no increase in lipid oxidation when cultured sea-bass fillets were subjected to a dose 8 times higher than the dose used in the present study.

### Total volatile basic nitrogen (TVB-N) and ammonia (NH_3_)

**Figure 4:**
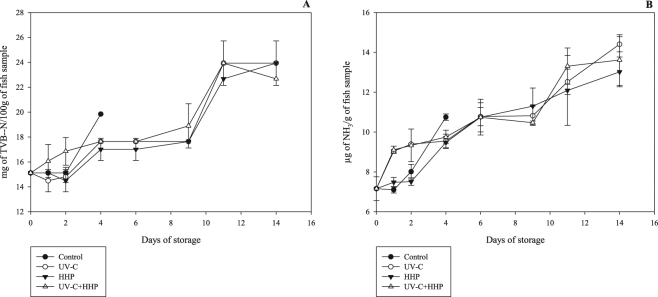
Total volatile basic nitrogen (TVB-N) and ammonia (NH_3_) in Nile tilapia (*Oreochromis niloticus*) fillets exposed to UV-C, HHP and UV-C+HHP processes under refrigeration storage (4 °C) for 14 days. Control – untreated fillets; UV-C – fillets treated with ultraviolet radiation type C (0.103 ± 0.002 J/cm^2^); HHP – fillets submitted to high hydrostatic pressure (220 MPa for 10 min at 25 °C); UV-C+HHP – fillets subjected to high hydrostatic pressure (220 MPa for 10 min at 25 °C) and ultraviolet radiation type C (0.103 ± 0.002 J/cm^2^). Error bars indicate the standard deviation of the mean (n = 5).

The increases (*P* ≤ 0.05) in TVB-N and ammonia levels in all treatments through the entire storage period are presented in Fig. 4. The initial levels were 15.12 ± 0.00 mg of TVB-N/100 g and 7.16 ± 0.60 µg of NH_3_/g of fish sample. These levels are in agreement with the findings of Ko *et al*.^43^ and Bottino *et al*.^40^, who investigated the freshness of Nile tilapia and hybrid tambacu, respectively, during refrigeration. In our study, both the TVB-N and ammonia levels increased during refrigerated storage of fish fillets, as previously observed^3–5,40,43^. TVB-N are nitrogenous compounds resulting from the action of spoilage microorganisms and enzymatic muscle activity. Ammonia is the main basic substance in this group, especially in freshwater fish such as Nile tilapia, which have low trimethylamine (TMA) levels due to lower trimethylamine oxide (TMAO) contents in their muscle tissue^51,52^. A limit of 25 mg of TVB-N/100 g of muscle tissue was established for rainbow-trout fillets by Giménez *et al*.^53^. In the present study, none of the fillets under any treatment reached this limit during the entire storage period (control = 19.85 ± 0.45 at day 4; HHP = 23.94 ± 1.78; UV-C = 23.94 ± 1.78; UV-C+HHP = 22.68 ± 0.00 at day 14), indicating that TVB-N was not an appropriate indicator of loss of quality, and this parameter was affected by the preservation treatments. This phenomenon was previously reported by Cyprian *et al*.^54^ and Monteiro *et al*.^3^ in refrigerated Nile-tilapia fillets.

No difference (*P* > 0.05) was found in TVB-N levels among treatments up to day 2 of storage. The control samples showed higher (*P* ≤ 0.05) TVB-N levels than the HHP, UV-C and UV-C+HHP samples on day 4; after this time, tilapia fillets treated with HHP and UV-C, either alone or together, showed similar (*P* > 0.05) TVB-N levels. Although UV-C and UV-C+HHP showed higher (*P* ≤ 0.05) ammonia levels than the UV-C and control samples up to day 2 of refrigerated storage, the tilapia fillets submitted to alternative conservation methods (HHP, UV-C and UV-C+HHP) reached about 10 µg NH_3_/g of muscle tissue on day 6, whereas the control samples reached this ammonia level on day 4. After this period, no difference (*P* > 0.05) was observed in ammonia levels among HHP, UV-C and UV-C+HHP samples. The initial TVB-N and ammonia levels could be associated with protein denaturation promoted by both the HHP and UV-C technologies^8,12^, followed by a compensatory effect due to prolonged bacterial doubling time over the storage period.

The increasing trend in the TVB-N and ammonia levels was slowed (*P* ≤ 0.05) in the HHP, UV-C and UV-C+HHP samples compared to their control counterparts throughout the storage period. This can be attributed to effect of the HHP and UV-C (alone or combined) on the mesophilic and Enterobacteria counts (Table 1), delaying the formation of ammonia and secondary and tertiary amines, which compose the total volatile basic nitrogen^5,41^. In accordance with our study, Ko *et al*.^43^ and Monteiro *et al*.^3^ observed increased TVB-N during refrigerated storage of Nile-tilapia fillets; and Monteiro *et al*.^4^ and Rodrigues *et al*.^5^ reported the same behavior for ammonia levels in Nile-tilapia and rainbow-trout fillets, respectively. Previous studies showed that TVB-N levels were lower in fish species treated with HHP than in untreated fish during refrigerated storage^22,44^, agreeing with our results. A trend toward delayed production of TVB-N and ammonia in freshwater fish species stored under refrigeration and exposed to UV-C was also observed by Bottino *et al*.^40^ and Rodrigues *et al*.^5^.

### Biogenic amines

**Figure 5:**
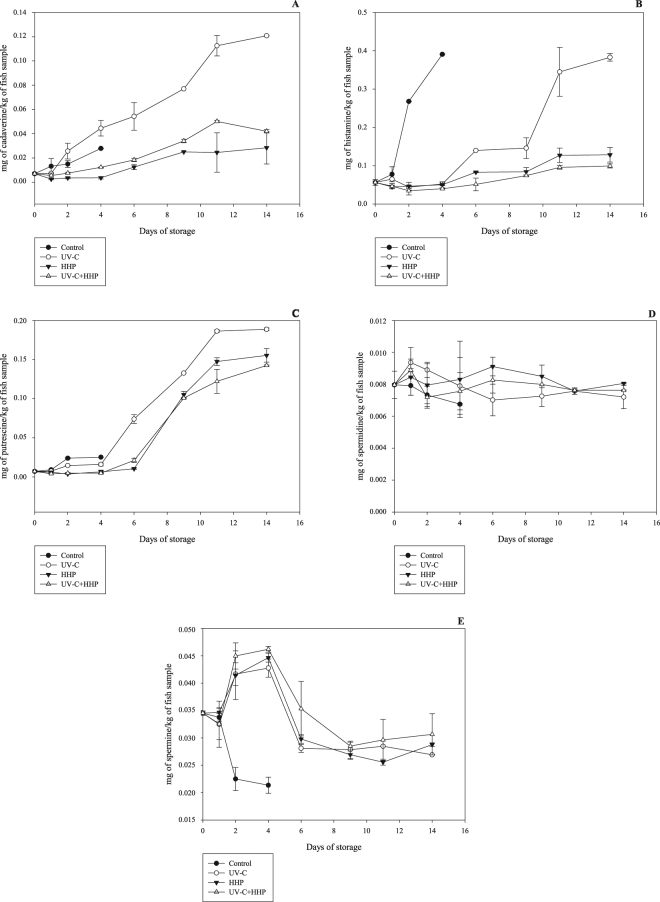
Levels of cadaverine (**A**), histamine (**B**), putrescine (**C**), spermidine (**D**) and spermine (**E**) in Nile tilapia (*Oreochromis niloticus*) fillets exposed to UV-C, HHP and UV-C+HHP processes under refrigeration storage (4 °C) for 14 days. Control – untreated fillets; UV-C – fillets treated with ultraviolet radiation type C (0.103 ± 0.002 J/cm^2^); HHP – fillets submitted to high hydrostatic pressure (220 MPa for 10 min at 25 °C); UV-C + HHP – fillets subjected to high hydrostatic pressure (220 MPa for 10 min at 25 °C) and ultraviolet radiation type C (0.103 ± 0.002 J/cm^2^). Error bars indicate the standard deviation of the mean (n = 5).

Histamine, cadaverine and putrescine increased (*P* ≤ 0.05), while spermidine remained constant (*P* > 0.05) and spermine decreased (*P* ≤ 0.05) during the storage period at 4 °C in all treatments (Fig. 5A–E). The accumulation of biogenic amines during refrigerated storage is related to the action of spoilage bacteria with positive decarboxylase activity, and therefore can be used as an indicator of quality loss^55^. The polyamines spermine and spermidine serve as sources of nitrogen for microorganisms, and are produced from putrescine^56^, reinforcing the results of this study.

Regulatory limits for histamine are 200 mg/kg for scombroid fish and 400 mg/kg for fish products, as set by the European Commission^57^; 500 mg/kg for fish, set by the FDA^58^; and 100 mg/kg for scombroid fish, set by the Brazilian federal government^59^. In our study, histamine levels remained below these limits in all treatments. Spermine and spermidine were not good indicators of quality loss, as also observed in other fish species including Nile tilapia^21,60^.

Control samples showed higher (*P* ≤ 0.05) histamine and putrescine levels than UV-C, HHP and UV-C+HHP over the storage period. From day 2 to day 14, putrescine and cadaverine levels were higher (*P* ≤ 0.05) in the UV-C-treated samples than the HHP and UV-C+HHP samples. After day 4, the HHP and UV-C+HHP samples showed lower (*P* ≤ 0.05) histamine, cadaverine and putrescine levels compared to UV-C samples. No difference (*P* > 0.05) was observed in spermidine content among all treatments, while both UV-C and HHP, alone or combined, increased (*P* ≤ 0.05) spermine levels until day 4 of storage. After this period, no difference (*P* > 0.05) was detected among the treatments. In general, both the UV-C and HHP methods cause DNA alterations and delay the formation of biogenic amines during storage^12,38^. The production of biogenic amines depends mainly on the microbiota (type and load) and the availability of precursor amino acids^55^; in addition, the effects of UV-C and HHP treatments vary according to the food matrix and processing conditions^7,14^. Information about the effects of UV-C and HHP on the formation of biogenic amines in muscle tissue of different fish species is sparse. However, UV-C radiation causes proteolysis, increasing the availability of free amino acids and thereby favoring the formation of some biogenic amines^12^. The increase of free amino acids after HHP treatment was also described^61^, associated with HHP-induced denaturation, which favors enzyme/substrate binding^62^. Moreover, UV-C radiation catalyzes the production of Fe^3+^, resulting in oxidative decarboxylation of amino acids^63,64^. In agreement with our findings, Rodrigues *et al*.^5^ observed similar patterns for cadaverine and putrescine levels in refrigerated rainbow-trout fillets treated with UV-C. Monteiro *et al*.^21^ reported that UV-C light increased the initial levels of histamine, cadaverine and putrescine, followed by a delay in their formation during refrigerated storage of Nile-tilapia fillets. Matejková *et al*.^65^ observed that HHP reduced putrescine and cadaverine levels during storage of vacuum-packed trout flesh, with no changes in the spermine and spermidine levels. Likewise, spermidine and spermine levels were not influenced by HHP, and only the putrescine content decreased during storage of pike flesh (*Esox lucius*)^66^.

## Conclusion

UV-C, HHP and UV-C+HHP did not affect the lag phase in the bacterial groups evaluated in this study; however, the treatments delayed bacterial growth and production of TVB-N, NH_3_ and biogenic amines, extending the shelf life of the tilapia fillets by 10 days under refrigerated storage considering the TAMC counts. However, cadaverine formation increased under the UV-C treatment. Lipid oxidation was not influenced by UV-C radiation, and decreased by HHP and UV-C+HHP. Based on these findings, HHP (220 MPa for 10 min at 25 °C) alone or in combination with UV-C radiation (0.103 ± 0.002 J/cm^2^) has more potential benefits for tilapia fillets preservation. Further studies are required considering sensory and instrumental characteristics related to flavor, visual appearance and texture modifications.
